# Long-term, single-center study comparing open and laparoscopic procedures for congenital midureteral obstruction in children

**DOI:** 10.1007/s00383-023-05494-y

**Published:** 2023-06-03

**Authors:** Guanglun Zhou, Man Jiang, Jianchun Yin, Xiaodong Liu, Junjie Sun, Shoulin Li

**Affiliations:** 1 Department of Urology and Laboratory of Pelvic Floor Muscle Function, Shenzhen Children’s Hospital, Futian District, Shenzhen, 518000 Guangdong People’s Republic of China; 2 Department of Infectious Diseases, Department of Urology and Laboratory of Pelvic Floor Muscle Function, Department of Clinical Laboratory, Shenzhen Children’s Hospital, Shenzhen, China

**Keywords:** Midureteral obstruction, Laparoscopy, Ureteroureterostomy, Children, Treatment

## Abstract

**Purpose:**

To compare the safety and outcomes of open and laparoscopic procedures in the management of congenital midureteral obstruction in children (CMO).

**Methods:**

Between February 2008 and February 2022, a total of 18 patients underwent open ureteroureterostomy (OU group), and 26 underwent laparoscopic ureteroureterostomy (LU group). The operative time, postoperative hospital stay, hospital costs, postoperative complications, and success rates of the two groups were compared.

**Results:**

The median age of the patients was 59 months, with 29 patients presenting with asymptomatic hydronephrosis, 12 with intermittent abdominal pain, and 3 with flank mass. The median follow-up time was 42 months, and all patients were successfully treated surgically. The operative time and postoperative hospital stay in the LU group were shorter than those in the OU group (106.3 ± 21.4 vs. 85.8 ± 16.5 min, 11.6 ± 1.9 vs. 8.3 ± 1.7 days, respectively; *p* < 0.05). The OU group had two postoperative complications, both of which were classified as Clavien–Dindo grade II based on the Clavien–Dindo classification. One case of postoperative complication occurred in the LU group, which was classified as Clavien–Dindo Grade II. There was no significant statistical difference in complications between the two groups (*P* > 0.05).

**Conclusions:**

Our data showed that laparoscopic ureteroureterostomy is a safe and effective treatment for congenital midureteral obstruction in children, and provides several advantages, including fewer postoperative complications, shorter postoperative hospital stay, and a shorter operative time. Laparoscopic procedures should be the first choice for treating children with congenital midureteral obstructions.

## Introduction

Congenital midureteral obstruction (CMO) is a rare disease in children, and its incidence rate is unknown [1]. Moreover, due to its rarity, no clear guidelines exist for the diagnosis and treatment of this disease [2]. CMO is an independent clinical disease that should be distinguished from ureteropelvic junction obstruction (UPJO) and ureterovesical junction obstruction (UVJO) [2]. CMO is mainly a mechanical obstruction [2]. CMO may cause progressive obstruction of the upper urinary tract and decline in renal function, which often requires surgical intervention [3]. Compared with UPJO and UVJO, CMO cases need repair more urgently [2]. If CMO is not treated promptly, it may lead to irreversible renal failure [3]. The ultimate goal of surgical techniques for CMO should be to effectively relieve obstruction and protect renal function with minimal trauma, and to achieve fewer postoperative complications.

Open surgical repair has been the gold standard for the surgical correction of ureteral obstruction in the past decades. Kannaiyan et al. reported that three children with midureteral stenosis underwent open surgery to remove the ureteral stricture segment and ureteroureterostomy, and the postoperative effect was satisfactory [4]. Hwang et al. reported that seven children with midureteral stenosis underwent open ureteroureterostomy; all patients were successfully cured [5]. Although open ureteroureterostomy is an effective method for surgical correction of ureteral obstruction [4, 5], it may involve a larger surgical wound [3]. With the breakthrough of minimally invasive technology, laparoscopic ureteral reconstruction has occasionally been reported in the treatment of patients with CMO [2, 6–8]. Nezhat et al. first reported a case of adult laparoscopic ureteroureterostomy for ureteral obstruction in 1992 [7]. They subsequently reported 8 cases of adult laparoscopic ureteroureterostomy [8]. Until recently, only a few cases of midureteral obstruction in children have been reported using laparoscopy [2]. In 2014, Chandrasekharam et al. reported that seven children with congenital midureteral stenosis underwent laparoscopic surgery and were successfully repaired [2]. In theory, laparoscopic repair has advantages over open surgery, such as better surgical vision and less trauma [3]. However, there is a lack of a relatively large case study of laparoscopic surgery for CMO, and no reports exist on the comparison of open versus laparoscopic surgeries for CMO.

In this study, we analyzed and compared the safety and long-term outcomes of open and laparoscopic surgeries for CMO in order to identify a satisfactory surgical method. In addition, this study evaluated the diagnostic accuracy of preoperative examinations.

## Materials and methods

This retrospective study was approved by our institutional ethics review committee and written informed consent was obtained from all guardians before surgery.

**Fig. 1:**
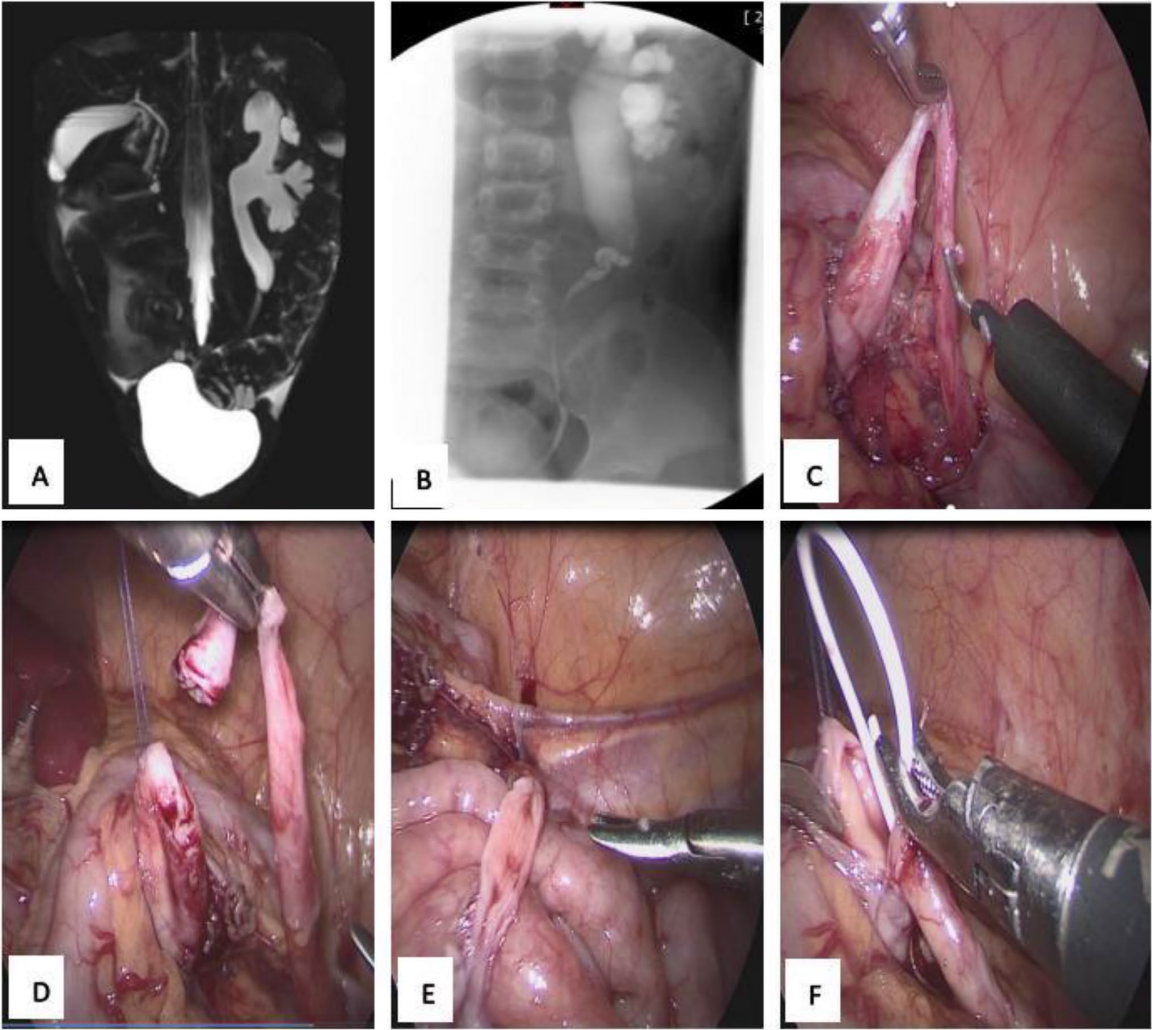
Image showing midureteral obstruction and laparoscopic ureteroureterostomy. **A** Magnetic resonance urography showing midureteral obstruction. **B** Retrograde pyelography showing midureteral obstruction. **C** Laparoscopy showed the obstructed segment ureter, the proximal dilated ureter, and the distal ureter. **D** The proximal ureter was pulled by suture, and then cut off the obstructed segment ureter. **E** The distal ureter was divided into a spade shape. **F** The double-J stent was placed through the anastomosis

We reviewed all patients with CMO who underwent primary surgery between February 2008 and February 2022 at the Shenzhen Children’s Hospital. The collected information included age, clinical features, preoperative and postoperative images (Fig. 1A, B), surgical details, outcomes, and postoperative complications. Patients with CMO coexisting with UVJO, UPJO, vesicoureteral reflux, or multiple ureteral stenoses were excluded from this study.

Ultrasound and intravenous pyelography were performed in all patients. Radioisotopic diethylenetriamine pentaacetic acid (DTPA) was administered to 41 patients. Magnetic resonance urography (MRU) was performed in 36 patients, and computed tomography urography (CTU) was performed in eight patients. Voiding cystourethrography was used to exclude the presence of vesicoureteral reflux in five children. Despite these efforts, the diagnosis remained uncertain in four who underwent retrograde pyelography and surgery at the same time.

The indication for surgery for CMO is one of the following: (1) progressive aggravation of hydronephrosis leading to a renal pelvis anteroposterior diameter of > 30 mm; (2) split renal function decreased by < 40%; (3) the presence of obvious clinical symptoms. A total of 44 patients with CMO were treated with open ureteroureterostomy (OU group, *n* = 18) or laparoscopic ureteroureterostomy (LU group, *n* = 26). The decision between the laparoscopic or open approaches was made based on the experience of the surgeon. Intraoperative and postoperative indicators, including operative time, postoperative hospital stay, hospital costs, postoperative complications, and follow-up outcomes, were compared between the OU and LU groups. The hospital costs were defined as all medical expenses incurred during and after surgery.

### Open techniques

Through the transverse incision in the middle abdomen of the lesion side, it was opened layer by layer, allowing an extraperitoneal approach to the ureter. The obstructed segment of the ureter was exposed and removed, and a catheter was inserted into the distal ureter. Water was injected to rule out the obstruction of the distal ureter. The distal ureter was divided into a spade shape to match the size of the proximal ureteral anastomosis, and ureteroureterostomy was performed. The suture process ensured that there was no tension at the anastomotic site. During the surgery, a double-J stent was placed through the anastomosis. One end of the stent was placed in the bladder and the other end was placed in the proximal ureter.

### Laparoscopic procedures

A transperitoneal laparoscopic approach with three ports was routinely used. The ureter was exposed, and the proximal ureter was pulled by suture. The obstructed segment of the ureter was then resected (Fig. 1C, D). The distal ureter was divided into a spade-like shape (Fig. 1E), and ureteroureterostomy was performed. The suture process ensured that there was no tension at the anastomotic site. The double-J stent was placed through the anastomosis (Fig. 1F). One end of the stent was placed in the bladder along the distal ureter, and the distal ureter was checked for obvious resistance. Then, the bladder was pressed to cause urine to flow back to the proximal end of the stent through the stent lumen, and urine flowed out of the stent through the lateral orifice, thus confirming that the distal stent tube is placed in the bladder. The other end of the stent was placed in the proximal ureter. In both open and laparoscopic procedures, a peritoneal drainage tube was placed near the anastomosis and removed after the drainage stopped. The Foley catheter was reserved for 5–7 days postoperatively. All resected specimens were subjected to a histological examination. The double-J stent was removed under anesthesia approximately four weeks after the operation.

### Follow-up

All patients were required to have a follow-up visit 3, 6, and 12 months after surgery and every year thereafter. The follow-up included physical examination and ultrasound examination of the urinary system. Surgical success was defined as an improvement of hydronephrosis (the renal pelvis anterior–posterior diameter at the last follow-up is smaller than that of the pre-surgery) or elimination of symptomatic obstruction.

### Statistical analysis

SPSS version 23.0 (IBM, New York, NY, USA) was used for statistical analysis of the data. Continuous data were analyzed using Student’s *t* test, and categorical variables were evaluated using the Chi-square test or Fisher’s exact test. Statistical significance was set at *P* < 0.05.

## Results

A total of 44 children were included in the study: 29 presented with asymptomatic hydronephrosis (19 cases were diagnosed prenatally), 12 with intermittent abdominal pain (2 cases had urinary tract infection), and 3 with flank mass. Among these patients, six had associated abnormalities, including 3 cases of contralateral multicystic dysplastic kidney, 2 cases of contralateral renal dysplasia, and 1 case of isolated kidney. All patients were confirmed to have CMO by surgery, including 33 cases of congenital midureteral stenosis, 6 cases of congenital midureteral polyps, 3 cases of congenital midureteral septum or valve, and 2 cases of retrocaval ureter. In children with congenital midureteral stenosis, the median length of the stricture segment was 0.8 cm (range, 0.4–1.8 cm). Preoperative ultrasound showed CMO in 18 cases (18/44, 40.9%); CMO was confirmed in 33 cases (33/44, 75%) by intravenous pyelography, in 33 cases (33/36, 91.7%) by MRU, in 7 cases (7/8, 87.5%) by CTU, and in 4 cases by retrograde pyelography. DTPA showed that 37 children had split renal function of < 40%.

The median age at operation in the OU group was 57 months, compared to 60 months in the LU group. The operation time and postoperative hospital stay in the LU group were lower than those in the OU group (*p* < 0.05); however, the operation cost in the LU group was higher than that in the OU group (*p* < 0.05). There were no conversions to open surgery in the LU group. The OU group had 2 postoperative complications (11.1%), both of which were classified as Clavien–Dindo grade II based on the Clavien–Dindo classification. One case of postoperative complication occurred in the LU group, which was classified as Clavien–Dindo Grade II. There was no significant statistical difference in complications between the two groups (*p* > 0.05). Children with postoperative complications were treated and discharged after recovery. The clinical characteristics and outcomes of the two groups are summarized in Tables 1 and 2.

**Table 1:** Clinical characteristics, outcomes of the open ureteroureterostomy and laparoscopic ureteroureterostomy groups

Observations	Open ureteroureterostomy	Laparoscopic ureteroureterostomy	*P* value
No. of patients	18	26	
Median age (months)	57 (range, 5–146)	60 (range, 4–175)	0.83
Sex			0.55
Male	12 (66.7%)	15 (57.7%)	
Female	6 (33.3%)	11 (42.3%)	
Side			0.77
Left	11 (61.1%)	17 (65.4%)	
Right	7 (38.9%)	9 (34.6%)	
Symptoms			0.96
Hydronephrosis	12 (66.7%)	17 (65.4%)	
Abdominal pain	5 (27.8%)	7 (26.9%)	
Flank mass	1 5.5%)	2 (7.7%)	
Etiology			0.48
Midureteral stenosis	14 (77.8%)	19 (73.1%)	
Midureteral polyp	2 (11.1%)	4 (15.4%)	
Retrocaval ureter	0 (0%)	2 (7.7%)	
Midureteral septum or valve	2 (11.1%)	1 (3.8%)

**Table 2:** Clinical outcomes of the open ureteroureterostomy and laparoscopic ureteroureterostomy groups

Observations	Open ureteroureterostomy	Laparoscopic ureteroureterostomy	*P* value
No. of patients	18	26	
Operation time (minutes)	106.3 ± 21.4	85.8 ± 16.5	< 0.01
Postoperative hospital stay (days)	12.2 ± 1.9	8.3 ± 1.7	< 0.01
Operation and postoperative hospital expenses (RMB)	11,010.3 ± 627.6	17,596.8 ± 516.2	< 0.01
Follow-up (months)	46 (10–120)	41 (6–78)	0.06
Success rate	18 (100%)	26 (100%)	0.33

The median follow-up time was 42 months, ranging from 6 to 120 months (10–120 months in the OU group and 6–78 months in the LU group). One patient in the LU group began to continuously drip urine 17 days after the operation, and the double-J tube was removed in advance because an ultrasound showed double-J tube displacement. Both the OU and LU groups achieved satisfactory success rates (100% vs. 100%, *p* > 0.05). Symptomatic obstruction was relieved or the hydronephrosis significantly improved in all children, and no recurrence of ureteral polyps was found.

## Discussion

In this study, our data suggested that laparoscopic ureteroureterostomy is a safe and effective treatment for CMO in children and has advantages over open surgery, including fewer postoperative complications, good cosmetic results.

At present, there are no comparative studies on laparoscopic and open procedures for the treatment of midureteral obstructions in children. Although open repair is considered the gold standard method, it may involve severe trauma, complications, and a long hospital stay [3]. The data of this study show that compared with open surgery, laparoscopic ureteroureterostomy not only achieves satisfactory results but also has a short operation time and postoperative hospital stay. In the LU group, the postoperative complications were less than those in the OU group, with only one case of urinary tract infection. The incision size required for the laparoscopic procedure of CMO is significantly smaller than that of the open procedure, which is beneficial to reduce postoperative pain, lessen the scarring, and lower wound infection. In 2016, Lu et al. used laparoscopic ureteroureterostomy to treat nine children with midureteral obstruction, with satisfactory surgical and cosmetic outcomes, and few postoperative complications [1]. In addition, for open surgery to treat CMO, the length of operation time mainly depends on the following factors: the time of incision and suture of the wound, the time of finding the ureteral obstruction segment and the time of ureteroureterostomy. The time of laparoscopic operation may mainly depend on ureteroureterostomy. Transabdominal laparoscopic surgery can provide more operative space, which is conducive to intraperitoneal suture and reduces the risk of bleeding, and can better avoid the occurrence of urine leakage [9, 10]. For children with midureteral obstruction, the length of the obstruction is usually short, which ensures that there is no tension during the ureteroureteral anastomosis. Compared with open procedure, we found that the length of postoperative hospital stay for CMO was shorter. This may be due to the smaller wound from laparoscopic procedures, less postoperative pain, short nursing time, and less anxiety of the guardian [6]. However, compared to open reconstruction, laparoscopic surgery has increased hospitalization costs. In this study, we did not use a retroperitoneal laparoscopic approach to treat the patients. Although this technique can avoid abdominal invasion, the operative space in children may be relatively limited [1].

CMO can lead to proximal ureteral dilatation, which is usually accompanied by hydronephrosis [6]. Ultrasound is a common imaging method used to detect urinary system hydrops in children. However, ultrasound has poor ability to predict the independent diagnosis of CMO in children. Arlen et al. reported that only 42% of patients with hydroureters were diagnosed using ultrasound [11]. In our study, the accuracy of the preoperative ultrasound diagnosis of ureteral obstruction was not high. Intravenous pyelography is a relatively accurate imaging technique for diagnosing upper urinary tract obstruction [6]. Meng et al. reported that the accuracy of intravenous pyelography for the diagnosis of ureteral stenosis was > 70% [6]. Similarly, this study showed that 75% of children with CMO were diagnosed using intravenous pyelography. For the diagnosis of CMO, MRU can provide satisfactory imaging [11]. In this study, the preoperative accuracy of MRU for the diagnosis of CMO was high. Lu et al. reported that the accuracy of MRU in diagnosing midureteral obstruction in 13 patients was as high as 100% [1]. It can provide useful information when a ureteral obstruction is suspected [11]. For a few patients who could not complete the MRU examination, CTU examination was performed at our institution. However, patients who still had difficulties in diagnosis after the above examinations underwent retrograde angiography to confirm the diagnosis. Retrograde pyelography can accurately determine the location and length of CMO and evaluate its severity [5]. Smith et al. considered that patients suspected of having CMO should undergo routine retrograde pyelography due to the high misdiagnosis rate of CMO [12]. However, this is not only an invasive examination but also a possible risk factor for iatrogenic ureterovesical junction obstruction, urinary tract infection, and ureteral injury [4]. Based on the data of this study, we recommend routine preoperative imaging of these children using ultrasound and intravenous pyelography. When the diagnosis is difficult, further MRU should be performed, and retrograde pyelography should be the final imaging method before reconstruction surgery. In this study, most of the asymptomatic hydronephrosis was diagnosed prenatally. For prenatal hydronephrosis, a meticulous postnatal evaluation is mandatory to develop the next management plan. During follow-up, the need for surgical intervention is determined based on the course of hydronephrosis in time and the impairment of renal function.

The placement of double-J ureteral stents is an important procedure in ureteral reconstructive surgery. Several methods of stent placement have been proposed. Some urologists prefer to use a cystoscope to insert a stent after surgery [13]. Lu et al. used a cystoscope to retrogradely insert double-J type stents into the renal pelvis when half of the ureteral anastomotic suture was completed [1]. Li et al. [14] and Chen et al. [15] reported having used two sections of ureteral catheter to insert the double-J ureteral stent through the lateral hole as a guide wire and placed the two ends of the stent tube into the bladder and renal pelvis, respectively. The above procedure is intended to place the two ends of the double-J stents into the renal pelvis and bladder to avoid displacement of the stent tubes. In this study, we placed the stent through the anastomosis, with one end of the stent first placed in the bladder and the other end of the stent placed in the proximal ureter instead of the renal pelvis. Our data showed that this method is less prone to displacement of the stent tube and is easy to operate.

This study had some limitations. Firstly, this was a retrospective study. The imaging method of follow-up is mainly ultrasound, which lacks the ability to accurately assess renal function. Second, our center does not use a robot-assisted approach to treat CMO, the learning curve of robot-assisted technology for treating CMO may be shorter. Despite these limitations, this is the first and largest series of studies comparing open and laparoscopic procedures for CMO in children.

## Conclusion

Laparoscopic ureteroureterostomy is a safe and effective treatment for CMO in children and has several advantages, including fewer postoperative complications, good cosmetic results, short postoperative hospital stay, and shorter operating time. Laparoscopic procedures should be the first choice for children with congenital midureteral obstructions. We recommend routine preoperative imaging, using ultrasound and intravenous pyelography, in children with CMO; when the diagnosis is difficult, further MRU should be performed.

## Data Availability

The datasets used and/or analyzed during the current study are available from the corresponding author on reasonable request.

## References

[1] Lu LS, Bi YL, Wang X, Ruan SS (2017). Laparoscopic resection and end­-to-end ureteroureterostomy for midureteral obstruction in children[J]. J Laparoendosc Adv Surg Tech A.

[2] Chandrasekharam VVS (2015). Laparoscopic repair of congenital midureteric strictures in infants and children. J Pediatr Surg.

[3] Tyritzis SI, Wiklund NP (2015). Ureteral strictures revisited…trying to see the light at the end of the tunnel: a comprehensive review. J Endourol.

[4] Kannaiyan L, Karl S, Mathai J, Chacko J, Sen S (2009). Congenital ureteric stenosis: a study of 17 children. Pediatr Surg Int.

[5] Hwang AH, Mcaleer IM, Shapiro E, Miller OF, Krous HF, Kaplan GW (2005). Congenital mid ureteral strictures. J Urol.

[6] Meng Z, Lin D, Wang G, Qu YC, Sun N (2021). Congenital midureteral stenosis in children: a 13-year retrospective study based on data from a large pediatric medical center. BMC Urol.

[7] Nezhat C, Nezhat F, Green B (1992). Laparoscopic treatment of obstructed ureter due to endometriosis by resection and ureteroureterostomy: a case report. J Urol.

[8] Nezhat CH, Nezhat F, Seidman D, Nezhat C (1998). Laparoscopic ureteroureterostomy: a prospective follow-up of 9 patients. Prim Care Update Ob Gyns.

[9] Hostiuc S, Rusu MC, Negoi I, Grigoriu M, Hostiuc M (2019). Retrocaval ureter: a meta-analysis of prevalence. Surg Radiol Anat.

[10] Tamhankar AS, Savalia AJ, Sawant AS, Pawar PW, Kasat GV, Patil SR (2017). Transperitoneal laparoscopic repair of retrocaval ureter: our experience and review of literature. Urol Ann.

[11] Arlen AM, Kirsch AJ, Cuda SP, Little SB, Jones RA, Grattan-Smith JD, Cerwinka WH (2014). Magnetic resonance urography for diagnosis of pediatric ureteral stricture. J Pediatr Urol.

[12] Smith BG, Metwalli AR, Leach J, Cheng EY, Kropp BP (2004). Congenital midureteral stricture in children diagnosed with antenatal hydronephrosis. Urology.

[13] Bhandarkar DS, Lalmalani JG, Shivde S (2003). Laparoscopic ureterolysis and reconstruction of a retrocaval ureter. Surg Endosc.

[14] Li HZ, Ma L, Qi L, Shi TP, Wang BJ, Zhang X (2010). Retroperitoneal laparoscopic ureteroureterostomy for retrocaval ureter: report of 10 cases and literature review. Urology.

[15] Chen Z, Chen X, Wu ZH, Luo YC, Li NN (2011). Treatment of retrocaval ureter by retroperitoneal laparoscopic ureteroureterostomy: experience on 12 patients. J Laparoendosc Adv Surg Tech A.

